# Changes to Racial Disparities in Readmission Rates After Medicare’s Hospital Readmissions Reduction Program Within Safety-Net and Non–Safety-Net Hospitals

**DOI:** 10.1001/jamanetworkopen.2018.4154

**Published:** 2018-11-02

**Authors:** Krisda H. Chaiyachati, Mingyu Qi, Rachel M. Werner

**Affiliations:** 1 Division of General Internal Medicine, University of Pennsylvania Perelman School of Medicine, Philadelphia; 2 Corporal Michael J. Crescenz Veterans Affairs Medical Center, Philadelphia, Pennsylvania; 3 Leonard Davis Institute of Health Economics, University of Pennsylvania, Philadelphia

## Abstract

**Importance:**

Although readmission rates are declining under Medicare’s Hospital Readmissions Reduction Program (HRRP), concerns remain that the HRRP will harm quality at safety-net hospitals because they are penalized more often. Disparities between white and black patients might widen because more black patients receive care at safety-net hospitals. Disparities may be particularly worse for clinical conditions not targeted by the HRRP because hospitals might reallocate resources toward targeted conditions (acute myocardial infarction, pneumonia, and heart failure) at the expense of nontargeted conditions.

**Objective:**

To examine disparities in readmission rates between white and black patients discharged from safety-net or non–safety-net hospitals after the HRRP began, evaluating discharges for any clinical condition and the subsets of targeted and nontargeted conditions.

**Design, Setting, and Participants:**

Cohort study conducting quasi-experimental analyses of patient hospital discharges for any clinical condition among fee-for-service Medicare beneficiaries from 2007 to 2015 after controlling for patient and hospital characteristics. Changes in disparities were measured within safety-net and non–safety-net hospitals after the HRRP penalties were enforced and compared with prior trends. These analyses were then stratified by targeted and nontargeted conditions. Analyses were conducted from October 1, 2017, through August 31, 2018.

**Main Outcomes and Measures:**

Trends in 30-day readmission rates among white and black patients by quarter and differences in trends across periods.

**Results:**

The study sample included 58 237 056 patient discharges (black patients, 9.8%; female, 57.7%; mean age [SD] age, 78.8 [7.9] years; nontargeted conditions, 50 372 806 [86.5%]). Within safety-net hospitals, disparities in readmission rates for all clinical conditions widened between black and white patients by 0.04 percentage point per quarter in the HRRP penalty period (95% CI, 0.01 to 0.07; *P* = .01). This widening was driven by nontargeted conditions (0.05 percentage point per quarter [95% CI, 0.01 to 0.08]; *P* = .006), whereas disparities for the HRRP-targeted conditions did not change (with an increase of 0.01 percentage point per quarter [95% CI, −0.07 to 0.10]; *P* = .74). Within non–safety-net hospitals, racial disparities remained stable in the HRRP penalty period across all conditions, whether the conditions were HRRP-targeted or nontargeted.

**Conclusions and Relevance:**

Findings from this study suggest that disparities are widening within safety-net hospitals, specifically for non–HRRP-targeted conditions. Although increases in racial disparities for nontargeted conditions were modest, they represent 6 times more discharges in our cohort than targeted conditions.

## Key Points

**Question:**

How have racial disparities in readmission rates between black and white patients changed within safety-net and non–safety-net hospitals after Medicare’s Hospital Readmissions Reduction Program (HRRP) began enforcing financial penalties in 2012?

**Findings:**

In this cohort study of Medicare data comprising 58.2 million hospital patients discharged from 2007 to 2015, black patients had worsening readmission rates in safety-net hospitals, specifically among clinical conditions not targeted by the HRRP, whereas differences among targeted conditions were stable. Within non–safety-net hospitals, racial disparities did not change for patients discharged with targeted or nontargeted conditions.

**Meaning:**

This study’s findings suggest that racial disparities may have widened substantially after the implementation of the HRRP for discharges within safety-net hospitals among nontargeted conditions.

## Teaser

This cohort study of Medicare data from 2007 to 2015 examines disparities in readmission rates between white and black patients discharged from safety-net or non–safety-net hospitals after initiation of the Hospital Readmissions Reduction Program (HRRP).

## Introduction

In October 2012, Medicare began financially penalizing hospitals with higher-than-expected 30-day readmission rates for select, targeted clinical conditions (acute myocardial infarction, pneumonia, and heart failure) under the Hospital Readmissions Reduction Program (HRRP).^1^ Since the announcement of this policy, readmission rates in the United States have declined for HRRP-targeted conditions^2,3,4^ and, to a lesser degree, for nontargeted conditions.^5^ Despite the perceived success of the HRRP, concerns remain that safety-net hospitals could be harmed by HRRP penalties.^6^

Hospital-based financial incentives may exacerbate health care disparities, particularly if they penalize institutions that disproportionately serve minority patients or reward hospitals that avoid minority patients.^7,8,9,10^ In the case of readmissions, black patients are more likely to be readmitted compared with white patients,^11^ safety-net hospitals care for a larger proportion of black patients than non–safety-net hospitals,^12^ and safety-net hospitals have higher readmission rates compared with non–safety-net hospitals.^13,14^ Indeed, the HRRP has reduced Medicare payments to safety-net hospitals by 1% to 3%.^15,16,17^ There has been some concern that, if payments are reduced, safety-net hospitals will have less revenue to invest in quality-improvement programs, such as reducing readmissions. Quality-improvement programs may have a larger positive outcome among black patients,^18,19,20,21^ for whom there was more room for improvement. As a result, we might observe widening racial differences in readmission rates within safety-net hospitals. Furthermore, because the HRRP targets a small group of conditions, persistently penalizing safety-net hospitals may force shifts in scarce resources (eg, social services or material goods that could prevent readmissions) toward conditions targeted by the HRRP at the expense of nontargeted conditions. Disparities for nontargeted conditions may be worsening even as disparities for targeted conditions decrease or stabilize.

Recent research has evaluated the association of HRRP implementation with racial disparities across all hospitals and found that racial differences in readmission rates between black and white patients did not worsen for the HRRP-targeted conditions (acute myocardial infarction, heart failure, and pneumonia) after the HRRP began enforcing penalties.^4^ Furthermore, changes in readmission rates were similar across hospitals with varying proportions of black patients. However, it remains unknown whether disparities between black and white patients differed within safety-net and non–safety-net hospitals, or whether findings differ by clinical condition, HRRP-targeted or not.

Thus, the objective of this study was to measure changes in disparities within safety-net and non–safety-net hospitals after the HRRP penalties were enforced and to compare them with trends prior to enforcement. We then stratified these analyses by HRRP-targeted and nontargeted conditions.

## Methods

### Data Sources

We used 2007 to 2015 data from 3 sources: (1) 100% Medicare Provider Analysis and Review files and the Medicare Beneficiary Summary File, which include demographic data, enrollment data, and hospital claims for all fee-for-service Medicare beneficiaries; (2) Medicare Provider of Services files, which include characteristics of Medicare-certified hospitals; and (3) the American Hospital Association Annual Survey of Hospitals, which includes data on the number of Medicaid discharges at each hospital. Medicare routinely collects data on race as part of their claims file. This study was approved by the institutional review board at the Perelman School of Medicine at the University of Pennsylvania, Philadelphia, which waived the need for informed consent because this was a retrospective study with minimal risk for loss of patient confidentiality, and the study could not have been carried out if the informed consent was required. The study was reported in accordance with the Strengthening the Reporting of Observational Studies in Epidemiology (STROBE) reporting guideline.

### Study Sample

A cohort was created of Medicare fee-for-service beneficiaries discharged from acute-care hospitals from January 1, 2007, through September 30, 2015, with any clinical condition. Inclusion criteria were patients 65 years or older who survived their hospitalization and were identified in the data set as either black or white (95% of all discharges). Previous studies indicate the racial categorization of patients within Medicare data to be valid.^22,23,24^ Following the HRRP rules, exclusion criteria were Medicare beneficiaries who were not enrolled in fee-for-service Medicare for 1 year prior to hospitalization and the month after hospital discharge, were discharged against medical advice or to hospice, and for whom the primary reason for hospitalization was a psychiatric condition, rehabilitation, or medical cancer treatment.^25^

### Study Variables

The primary outcome was whether a beneficiary had an unplanned readmission for any clinical condition within 30 days of hospital discharge using Medicare’s definition.^25^ The independent variables of interest were the race of the patient (white or black), the quarter in which the patient was discharged, and whether or not the discharging hospital was a safety-net hospital, that is, hospitals in the top quartile of Medicaid discharges as a proportion of all discharges, by state, measured the first year the hospital had a discharge.^26^

For risk-adjusted readmission rates, we adjusted for discharge characteristics (ie, patient age, sex, 30 comorbidities defined by Medicare’s hospital readmission risk adjustment,^25^ and Medicare/Medicaid dual-eligible status) and hospital characteristics (ie, number of beds, profit status, teaching hospital status [indicated by having an allopathic or osteopathic residency program], rural location of hospital, and US Census–designated region^27^). Discharge characteristics were measured for each hospital discharge and, with the exception of safety-net status, hospital characteristics were measured annually. Safety-net status was measured once in the first year the hospital was observed in the data.

### Statistical Analysis

The goals were to estimate trends in readmission rates for white and black patients within safety-net and non–safety-net hospitals irrespective of the primary clinical condition, to estimate differential trends between races, and to compare the difference-in-differences of trends across 3 periods: (1) prior to the implementation of the Affordable Care Act (ACA) on April 1, 2010 (pre-ACA period); (2) after the ACA and before the HRRP penalties were enforced on October 1, 2012 (HRRP implementation period); and (3) after the HRRP enforced penalties (HRRP penalty period). These periods are based on prior research showing significant readmission rate declines during the implementation period.^2,5^

To calculate our estimates and compare differential trends across periods, the following linear spline regression model^28^ with hospital fixed effects was fitted:

E(Y_ijt_) = β_0_ + β_1_black_i_ + β_2_time_t_ + β_3_postACA_t_ + β_4_postHRRP_t_ + β_5_black_i_ × time_t_ + β_6_black_i_ × postACA_t_ + β_7_black_i_ × postHRRP_t_ + β_8_X_ijt_ + α_j_,

where E(Y_ijt_) is whether there was a readmission within 30 days of discharge for the *i*th discharge from the *j*th hospital during time quarter *t*; black is coded as 1 if the patient being discharged was black and 0 if the patient was white; time is the quarter of discharge beginning with the first quarter of 2007 (the beginning of the data set), ranging from 0 to 35; postACA is a quarter dummy variable that equaled 0 if the discharge was prior to the ACA (second quarter of 2010) and ranged from 0 to 21 if after the ACA; postHRRP is a quarter dummy variable that equaled 0 if the discharge was prior to the HRRP-enforced penalties (fourth quarter of 2012) and ranged from 0 to 11 if after the HRRP-enforced penalties; X_ijt_ are the patient and hospital covariates described above; and α_j_ is hospital fixed effects to account for time-invariant hospital factors. All regressions adjusted the SEs for clustering of patients within hospitals using Huber-White SEs.^29^

This resulted in the following estimates of trends in readmission rates by quarter: trend in readmission rates for white patients in the pre-ACA period (β_2_), for black patients in the pre-ACA period (β_2_ + β_5_), and the differences in trends between black patients relative to white patients in the pre-ACA period (β_5_); the trend in readmission rates for white patients in the HRRP implementation period (β_2_ + β_3_), for black patients in the HRRP implementation period (β_2_ + β_3_ + β_5_ + β_6_), and the differences in trends between black patients relative to white patients in the HRRP implementation period (β_5_ + β_6_); and the trend in readmission rates for white patients in the HRRP penalty period (β_2_ + β_3_ + β_4_), for black patients in the HRRP penalty period (β_2_ + β_3_ + β_4_ + β_5_ + β_6_ + β_7_), and the differences in trends for black patients relative to white patients in the HRRP penalty period (β_5_ + β_6_ + β_7_).

The difference-in-differences of these trends were further estimated in readmission rates. That is (1) the difference-in-differences of trends in readmission rates for black patients relative to white patients in the pre-ACA period relative to that difference in trends in the HRRP implementation period (β_6_); (2) the difference-in-differences of trends for black patients relative to white patients in the HRRP implementation period relative to that difference in trends in the HRRP penalty period (β_7_); and (3) the difference-in-differences of trends for black patients relative to white patients in the pre-ACA period relative to that difference in trends in the HRRP penalty period (β_6_ + β_7_). Negative estimations indicate that trends in disparities narrowed (suggesting relative improvements in disparities), whereas positive estimations indicate that trends in disparities widened (suggesting a relative worsening in disparities).

Finally, there was stratification based on the discharge condition: whether patients were discharged for HRRP-targeted (targeted conditions) (acute myocardial infarction, heart failure, or pneumonia) or non–HRRP-targeted conditions (nontargeted conditions). Separate regressions were run for each group of conditions and for safety-net and non–safety-net hospitals.

Analyses were performed with the use of Stata, version 14.1 (StataCorp). All hypothesis testing was conducted using a 2-sided, type I error rate of .05. Analyses were conducted from October 1, 2017, through August 31, 2018.

## Results

Our cohort included 58 237 056 discharges from 3871 hospitals (Table 1 and Table 2). These included 11 237 242 discharges (19.3%) from safety-net hospitals and 46 999 814 (80.7%) from non–safety-net hospitals, and 7 864 250 discharges (13.5%) for targeted conditions and 50 372 806 (86.5%) for nontargeted conditions. Among all discharges, 9.8% were of black patients, 57.7% were women, 16.8% were dually enrolled in Medicare and Medicaid, and the mean (SD) age was 78.8 (7.9) years. There was no difference across the 3 periods with regard to patient demographics or hospital characteristics (eTable 1 in the Supplement).

**Table 1.** Characteristics of Black and White Medicare Beneficiary Discharges From Safety-Net and Non–Safety-Net Hospitals, 2007-2015

Patient Characteristics	Hospitals				
	All	Safety-Net (n = 11 237 242 [19.3%])		Non–Safety-Net (n = 46 999 814 [80.7%])	
		White Patients	Black Patients	White Patients	Black Patients
Discharges, No. (%)	58 237 056 (100.0)	9 558 665 (85.1)	1 678 577 (14.9)	42 950 074 (91.4)	4 049 740 (8.6)
Clinical condition, No. (%)					
HRRP-targeted^a^	7 864 250 (13.5)	1 271 540 (13.3)	235 906 (14.1)	5 793 376 (13.5)	563 428 (13.9)
Nontargeted	50 372 806 (86.5)	8 287 125 (86.7)	1 442 671 (85.9)	37 156 698 (86.5)	3 486 312 (86.1)
Women, No. (%)	33 582 620 (57.7)	5 433 972 (56.8)	1 030 062 (61.4)	24 623 073 (57.3)	2 495 510 (61.6)
Dual-eligible for Medicare and Medicaid, No. (%)	9 800 534 (16.8)	1 682 263 (17.6)	722 360 (43.0)	5 781 363 (13.5)	1 614 472 (39.9)
Age, mean (SD), y	78.8 (7.9)	78.5 (7.9)	77.2 (8.0)	79.0 (7.9)	77.4 (8.0)

Abbreviation: HRRP, Hospital Readmissions Reduction Program.

^a^ The HRRP-targeted conditions included acute myocardial infarction, heart failure, and pneumonia.

**Table 2.** Characteristics of Safety-Net and Non–Safety-Net Hospitals With Medicare Beneficiary Discharges, 2007-2015

Hospital Characteristics	Hospitals, No. (%)		
	All (N = 3871)	Safety-Net (n = 824)	Non–Safety-Net (n = 3047)
Bed size, mean (SD), No.	210 (213)	259 (266)	196 (193)
Profit status^a^			
For profit	1100 (28.4)	173 (16.0)	927 (24.4)
Nonprofit	2041 (52.7)	421 (38.9)	1620 (42.6)
Public	482 (12.5)	164 (15.2)	318 (8.4)
Other	1261 (32.6)	324 (29.9)	937 (24.6)
Teaching hospital	1002 (25.9)	289 (26.7)	713 (23.4)
Rural hospital	1061 (27.4)	275 (33.4)	786 (25.8)
Geographic region			
Northeast	588 (15.2)	132 (16.0)	456 (15.0)
Midwest	858 (22.2)	160 (19.4)	698 (22.9)
South	1685 (43.5)	385 (46.7)	1300 (42.7)
West	740 (19.1)	147 (17.8)	593 (19.5)

^a^ Sum of columns is more than 100% because many hospitals (26%) changed their profit status at least once during the study period.

### Changes in Readmission Disparities Within Safety-Net and Non–Safety-Net Hospitals

In the first quarter of 2007, white and black patients discharged from safety-net hospitals had relatively higher risk-adjusted readmission rates (17.34% [95% CI, 17.24% to 17.45%] and 18.39% [95% CI, 18.16% to 18.61%], respectively) than those from non–safety-net hospitals (16.64% [95% CI, 16.59% to 16.70%] and 17.68% [95% CI, 17.53% to 17.84%], respectively) (Table 3). In most cases, readmission rates for black and white patients across safety-net and non–safety-net hospitals declined over the HRRP implementation period. Only black patients within safety-net hospitals and non–safety-net hospitals had higher readmission rates at the end of the study period compared with the previous time point.

**Table 3.** Risk-Adjusted Readmission Rates for Black and White Medicare Beneficiaries Discharges From Safety-Net and Non–Safety-Net Hospitals, 2007-2015^a^

Time Point^b^	Readmitted Patients, % (95% CI)^c^			
	Safety-Net Hospitals		Non–Safety-Net Hospitals	
	White Patients	Black Patients	White Patients	Black Patients
Start of sample (2007, Q1)	17.34 (17.24-17.45)	18.39 (18.16-18.61)	16.64 (16.59-16.70)	17.68 (17.53-17.84)
HRRP implemented (2010, Q2)	17.08 (17.00-17.16)	18.32 (18.12-18.53)	16.33 (17.37-17.61)	17.49 (17.37-17.61)
HRRP penalties enforced (2012, Q4)	15.72 (15.63-15.80)	15.91 (15.70-16.13)	14.95 (14.91-15.00)	15.65 (15.53-15.76)
End of sample (2015, Q3)	15.64 (15.52-15.77)	16.30 (16.04-16.56)	14.90 (14.84-14.96)	15.67 (15.52-15.84)

Abbreviations: HRRP, Hospital Readmissions Reduction Program; Q, quarter.

^a^ Risk-adjusted readmission rates and 95% CIs were estimated at key policy transition points between 2007 and 2015 using the linear spline multivariable linear regression models and the margins postestimation command in Stata, version 14.1 (StataCorp).

^b^ The start of the study sample began on January 1, 2007. The Affordable Care Act was implemented on April 1, 2010. The HRRP began enforcing penalties September 30, 2012. The study sample ended on September 30, 2015.

^c^ All estimates were adjusted for patient characteristics (ie, age, sex, 30 comorbidities defined by Medicare’s hospital readmission risk adjustment,^25^ and Medicare/Medicaid dual-eligible status^26^), hospital characteristics (ie, number of beds, profit status, teaching hospital status [indicated by having an allopathic or osteopathic residency program], rural location of hospital, and US Census–designated region^27^), and incorporated hospital fixed effects.

When measuring trends in disparities in readmission by periods (Table 4), we did not observe disparities narrowing between white and black patients in the pre-ACA period within safety-net hospitals (black-white difference in rates of decline of readmission rates changed 0.02 percentage point per quarter [95% CI, −0.01 to 0.04]; *P* = .22) or non–safety-net hospitals (difference, 0.01 percentage point per quarter [95% CI, −0.01 to 0.03]; *P* = .26). During the HRRP implementation period, black patients had a steeper decline in readmission rates than white patients in safety-net hospitals (−0.24 percentage point per quarter for black patients vs −0.14 percentage point per quarter for white patients), resulting in a narrowing of disparities in readmission rates by −0.11 percentage point per quarter (95% CI, −0.13 to −0.07; *P* < .001). Non–safety-net hospitals exhibited a smaller magnitude of narrowing of disparities in readmission rates (difference, −0.05 percentage point per quarter [95% CI, −0.07 to −0.03]; *P* < .001). In the HRRP penalty period, safety-net hospitals exhibited widening of readmission disparities (0.04 percentage point per quarter [95% CI, 0.01-0.07]; *P* = .01), whereas differences in readmission rates within non–safety-net hospitals remained stable (0.01 percentage point per quarter [95% CI, −0.01 to 0.03]; *P* = .43).

**Table 4.** Risk-Adjusted Differences in Readmission Trends Between Black and White Medicare Beneficiary Discharges by Study Period and Difference-in-Differences in Trends Between Study Periods Among Safety-Net and Non–Safety-Net Hospitals, 2007-2015

Period^a^	Safety-Net Hospitals (n = 11 237 242 Discharges)^b^				Non–Safety-Net Hospitals (n = 46 999 814 Discharges)^b^			
	White Patients	Black Patients	Difference (95% CI)	*P* Value	White Patients	Black Patients	Difference (95% CI)	*P* Value
**Difference in Readmission Trends Between Races**								
Pre-ACA	−0.02	0	0.02 (−0.01 to 0.04)	.22	−0.02	−0.02	0.01 (−0.01 to 0.03)	.26
HRRP implementation	−0.14	−0.24	−0.11 (−0.13 to −0.07)	<.001	−0.14	−0.18	−0.05 (−0.07 to −0.03)	<.001
HRRP penalty	−0.01	0.03	0.04 (0.01 to 0.07)	.01	0	0	0.01 (−0.01 to 0.03)	.43
**Difference in Racial Readmission Differences Between Periods**								
Pre-ACA vs HRRP implementation			−0.12 (−0.17 to −0.07)	<.001			−0.06 (−0.09 to −0.03)	<.001
HRRP implementation vs HRRP penalty			0.15 (0.09 to 0.20)	<.001			0.05 (0.02 to 0.09)	.001
HRRP penalty vs pre-ACA			0.03 (−0.01 to 0.06)	.18			0 (−0.02 to 0.02)	.90

Abbreviations: ACA, Affordable Care Act; HRRP, Hospital Readmissions Reduction Program.

^a^ The pre-ACA period began on January 1, 2007, and ended on March 31, 2010. The HRRP implementation period began on April 1, 2010, and ended on September 30, 2012. The HRRP penalty period began on October 1, 2012, and ended on September 30, 2015.

^b^ All estimates were adjusted for patient characteristics (ie, age, sex, 30 comorbidities defined by Medicare’s hospital readmission risk adjustment,^25^ and Medicare/Medicaid dual-eligible status^26^), hospital characteristics (ie, number of beds, profit status, teaching hospital status [indicated by having an allopathic or osteopathic residency program], rural location of hospital, and US Census–designated region^27^), and incorporated hospital fixed effects.

When comparing differences in trends in readmission rates between races across periods, the difference-in-differences (Table 4), disparities in readmission rates within safety-net hospitals narrowed more rapidly during the HRRP implementation period compared with the pre-ACA period (trends in readmission rates disparities declined faster by a relative −0.12 percentage point per quarter [95% CI, −0.17 to −0.07 for black patients compared with white patients; *P* < .001). However, comparing the HRRP implementation period with the HRRP penalty period, disparities in readmissions stopped improving (0.15 percentage point per quarter [95% CI, 0.09-0.20]; *P* < .001) but were not statistically different from the differences in trends in the pre-ACA period (0.03 percentage point per quarter [95% CI, −0.01 to 0.06]; *P* = .18). Within non–safety-net hospitals, the difference-in-differences of trends in readmission rates between races between periods were similar but of smaller magnitude relative to safety-net hospitals.

### Changes in Readmission Disparities for Targeted and Nontargeted Conditions

When comparing trends in disparities in readmission rates by period for targeted conditions, we observed the same trends in safety-net and non–safety-net hospitals (Figure, A and B; eTable 2 in the Supplement). There were no statistically significant differences in changing readmission rates between white and black patients in the pre-ACA period for safety-net hospitals. During the HRRP implementation period, black patients had a steeper decline in readmission rates than white patients, resulting in significant narrowing of disparities in both hospital types. During the HRRP penalty period, there were no statistically significant differences in trends.

**Figure.**
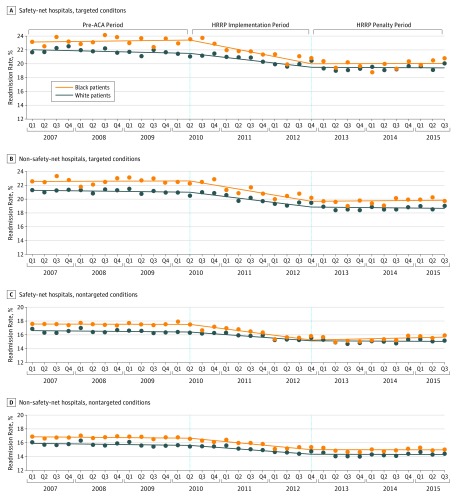
Risk-Adjusted Differential Trends in Readmission Rates Between Discharges of White and Black Patients Between 2007-2015 for Discharges by Safety-Net Hospital Status and Clinical Condition The pre–Affordable Care Act (ACA) period began on January 1, 2007, and ended on March 31, 2010. The Hospital Readmissions Reduction Program (HRRP) implementation period began on April 1, 2010, and ended on September 30, 2012. The HRRP penalty period began on October 1, 2012, and ended on September 30, 2015. Risk-adjusted readmission rates were calculated using a linear regression model containing the interaction between race and quarter and the “predict” postestimation command for each quarter. All models were adjusted for patient characteristics at discharge (ie, age, sex, 30 comorbidities defined by Medicare’s hospital readmission risk adjustment, and Medicare and Medicaid dual-eligible status), hospital characteristics (ie, number of beds, profit status, teaching hospital status [as indicated by having an allopathic or osteopathic residency program], rural location of hospital, and US Census–designated region), and incorporated hospital fixed effects. Trends in risk-adjusted readmission rates were calculated using a linear spline regression model for the 4 combinations of safety-net status and clinical condition. Recycled predictions were used with the “predict” postestimation command in Stata after each regression to estimate trends in readmission rates by race within each period: the pre-ACA period, the HRRP implementation period, and the HRRP penalty period. These models were adjusted for patient characteristics at discharge, hospital characteristics, and incorporated hospital fixed effects. Q indicates quarter.

When comparing trends in disparities by period for nontargeted conditions, trends in readmission rates were similar to targeted conditions in the pre-ACA and HRRP implementation periods (Figure, C and D; eTable 3 in the Supplement). However, during the HRRP penalty period, readmission disparities widened for nontargeted conditions within safety-net hospitals (0.05 percentage point per quarter [95% CI, 0.01-0.08]; *P* = .006), whereas disparities for the HRRP-targeted conditions did not change (with an increase of 0.01 percentage point per quarter [95% CI, −0.07 to 0.10]; *P* = .74). During the HRRP penalty period in non–safety-net hospitals, readmission disparities were stagnant for nontargeted conditions (0.00 percentage point per quarter [95% CI, −0.01 to 0.02]; *P* = .81), neither narrowing nor widening.

## Discussion

Disparities in readmission rates may be widening within safety-net hospitals after the enforcement of the HRRP penalties, particularly for conditions that are not targeted by the HRRP. In contrast, disparities in readmission rates were improving during the HRRP implementation phase within safety-net and non–safety-net hospitals, irrespective of the clinical condition.

Prior research found that trends in readmission rates did not differ between black and white patients after the HRRP penalties, specifically for targeted conditions.^4^ Secondarily, those observations were similar across hospitals with varying proportions of black patients. Additional studies have shown that readmission rates declined more quickly after the ACA was enacted and that these declines slowed after the HRRP penalties among targeted^2^ and, by a smaller magnitude, nontargeted conditions.^5^ This study adds to this literature by comprehensively evaluating the consequences of the HRRP penalties for racial disparities. First, the consequences of the HRRP within safety-net and non–safety-net hospitals were specifically studied. Second, financial penalties for readmissions may have potential spill-over consequences for nontargeted conditions, which were 6 times more common than targeted ones.

These findings, in part, tell a positive story about racial disparities. That is, racial disparities in readmission rates significantly improved after the implementation of the ACA within safety-net and non–safety-net hospitals, for targeted and nontargeted conditions. In the years prior to the ACA, Medicare began publicly releasing data on discharge planning and readmission rates.^30^ Then, with the passage of the ACA, the focus on reducing readmission rates became even more intense, partly in anticipation of the HRRP. This focus may have catalyzed the implementation and diffusion of care delivery improvements and organizational changes^18,19,20,21,31^ that have been shown to reduce readmission rates for targeted and nontargeted conditions. These changes may have resulted in greater changes among black patients, for whom there was more room for improvement or whose readmissions are more sensitive to discharge planning improvements, such as enhanced social services. Although prior work has shown significant reductions in readmission rates during the HRRP implementation period, both nationally^2^ and within minority-serving hospitals,^4^ the results in this study show that these improvements were seen across safety-net and non–safety-net hospitals.

However, the findings suggest that opportunities for improvement exist. At best, racial disparities in readmission rates remain and now appear stagnant within non–safety-net hospitals. It is unknown why readmission rates stopped declining once the HRRP was implemented. The significant gains in readmission rates made in the 2 years prior to the HRRP may make further reductions difficult. Readmission rates should not be too low because readmissions are sometimes a necessary part of high-quality care, and it is unknown how low is too low. The diverging trends for targeted and nontargeted conditions within safety-net hospitals are concerning findings. Because safety-net hospitals are persistently penalized,^15,16,17,32^ they may be reallocating limited financial resources toward organizational changes meant to improve the care for targeted conditions. Although recent evidence suggests that the overall financial performance of safety-net hospitals may not have been harmed under the HRRP,^33^ hospitals may still be concentrating their finances toward avoiding future penalties for targeted conditions. As a result, programs that may improve the quality of care for nontargeted conditions may become less well funded or deployed more narrowly for targeted conditions. These reallocated resources may better support the needs of black patients compared with white patients. Although the absolute magnitude of the disparities increase for nontargeted conditions is relatively small, nontargeted conditions are much more common. Thus, even small increases in disparities could have consequences for a larger portion of the population.

Nonetheless, these findings should be interpreted within the context of prior trends. Although non–safety-net hospitals experienced widening disparities in the HRRP penalty period, particularly for nontargeted conditions, these observed differences in trends are not different from the pre-ACA period, which did not have significant widening. Nevertheless, we may be observing the early phase of the HRRP’s longer-term consequences on readmission disparities between white and black patients.

Policy recommendations to minimize penalties for hospitals serving a higher proportion of nonwhite, low-income patients have largely focused on actuarial risk adjustments before determining penalties.^34,35,36^ These adjustments may be helpful but are likely insufficient because previous studies show that these adjustments may not improve reimbursements for safety-net hospitals.^37^ Further reductions in disparities will require an understanding of which hospital strategies lead to persistent reductions in readmission rates among vulnerable patients, including black patients, such that readmission rates for racial minorities are reduced to levels consistent with their white counterparts. Hospital-wide readmission measures under consideration by the Centers for Medicare & Medicaid Services and the National Quality Forum may force an equal spotlight onto targeted and nontargeted conditions, potentially mitigating differences in trends by clinical condition. However, hospital-wide readmission penalties are expected to penalize more safety-net hospitals and generate larger penalties.^38^ The findings of this study indicate that safety-net hospitals are particularly sensitive to penalties. With a hospital-wide readmission measure, they may treat readmissions for all conditions equally, but these findings suggest resources could shift away from other operational objectives and affect quality elsewhere.

### Limitations

This study has several limitations. The observational, nonexperimental study design limits the ability to make causal links between HRRP and the outcomes of interest. However, the use of quasi-experimental approaches using longitudinal data from all HRRP-eligible discharges allowed us to draw credible associations between the enforcement of the HRRP penalties and changes in trends for the disparities between white and black patients. Alternative definitions for *safety-net* may result in different findings; however, other commonly used definitions^37,39^ are likely colinear with the definition that was used. Alternative explanations for reductions in readmissions (such as changes in coding practices, which may, in part, explain reductions in readmission) cannot be excluded.^40^ Despite this possibility, there is no reason to believe that changes in coding practices occurred differently for black vs white patients and, therefore, are unlikely to affect the results. In addition, there is a possibility that unmeasured sociodemographic characteristics that differ between black and white patients may have affected our findings.

## Conclusions

Concerns regarding the unintended consequences of the HRRP on racial disparities within safety-net hospitals have been raised frequently. These findings provide evidence that disparities may be worsening, specifically for nontargeted conditions. However, the implementation period between the enactment of the ACA and the HRRP’s enforcement of penalties is a critical period of improved equity between races irrespective of the hospital’s safety-net status or the patient’s clinical condition. Discovering what contributed to these observed trends and how readmission penalties are influencing hospital behavior will help future efforts aimed at improving equity within health care.

## Notes

Supplement. **eTable 1.** Characteristics of Hospital Discharges Among Medicare Beneficiaries Discharged Between 2007-2015 Under Medicare's Hospital Readmissions Reduction Program (HRRP) and Characteristics of the Discharging Hospitals, by Study Periods **eTable 2.** Risk-Adjusted Trends, Differences in Trends Between Black and White Patients by Periods and Difference-in-Differences in Trends Between Periods, by Hospitals’ Safety-Net Status Among the HRRP Targeted Conditions **eTable 3.** Risk-Adjusted Trends, Differences in Trends Between Black and White Patients by Periods and Difference-in-Differences in Trends Between Periods, by Hospitals’ Safety-Net Status Among Conditions Not Targeted by the HRRP
