# Supplementary material for: Changes to Racial Disparities in Readmission Rates After Medicare’s Hospital Readmissions Reduction Program Within Safety-Net and Non–Safety-Net Hospitals
Source: JAMA Netw Open. 2018 Nov 2;1(7):e184154. doi: 10.1001/jamanetworkopen.2018.4154 (PMC6324411; doi:10.1001/jamanetworkopen.2018.4154)
Supplement: Supplement. — eTable 1. Characteristics of Hospital Discharges Among Medicare Beneficiaries Discharged Between 2007-2015 Under Medicare's Hospital Readmissions Reduction Program (HRRP) and Characteristics of the Discharging Hospitals, by Study Periods eTable 2. Risk-Adjusted Trends, Differences in Trends Between Black and White Patients by Periods and Difference-in-Differences in Trends Between Periods, by Hospitals’ Safety-Net Status Among the HRRP Targeted Conditions eTable 3. Risk-Adjusted Trends, Differences in Trends Between Black and White Patients by Periods and Difference-in-Differences in Trends Between Periods, by Hospitals’ Safety-Net Status Among Conditions Not Targeted by the HRRP [file jamanetwopen-1-e184154-s001.pdf]

Supplementary Online Content

Chaiyachati KH, Qi M, Werner RM. Changes to racial disparities in readmission rates after Medicare's Hospital Readmissions Reduction Program within safety-net and non–safety-net hospitals. *JAMA Netw Open.* 2018;1(7):e184154. doi:10.1001/jamanetworkopen.2018.4154

eTable 1. Characteristics of Hospital Discharges Among Medicare Beneficiaries Discharged Between 2007-2015 Under Medicare's Hospital Readmissions Reduction Program (HRRP) and Characteristics of the Discharging Hospitals, by Study Periods

eTable 2. Risk-Adjusted Trends, Differences in Trends Between Black and White Patients by Periods and Difference-in-Differences in Trends Between Periods, by Hospitals' Safety-Net Status Among the HRRP Targeted Conditions

eTable 3. Risk-Adjusted Trends, Differences in Trends Between Black and White Patients by Periods and Difference-in-Differences in Trends Between Periods, by Hospitals' Safety-Net Status Among Conditions Not Targeted by the HRRP

This supplementary material has been provided by the authors to give readers additional information about their work.

© 2018 Chaiyachati KH et al. *JAMA Network Open*.

eTable 1. Characteristics of Hospital Discharges Among Medicare Beneficiaries Discharged Between 2007-2015 Under Medicare's Hospital Readmissions Reduction Program (HRRP) and Characteristics of the Discharging Hospitals, by Study Periods

Characteristics	Pre-ACA	HRRP implementation	HRRP penalty
Patient characteristics			
Discharges, No.	23,348,877	16,616,892	18,271,287
Discharge location, No. (%)			
Safety-net	4,477,502 (19.2)	3,207,257 (19.3)	3,552,483 (19.4)
Non-safety-net	18,871,375 (80.8)	13,409,635 (80.7)	14,718,804 (80.6)
Reason for admission, No. (%)			
Targeted condition	3,205,034 (13.7)	2,181,492 (13.1)	2,477,724 (13.6)
Non-targeted condition	2,0143,843 (86.3)	14,435,400 (86.9)	15,793,563 (86.4)
Black, No. (%)	2,254,110 (9.7)	1,648,623 (9.9)	1,825,584 (10.0)
Female, No. (%)	13,548,867 (58.0)	9,599,375 (57.8)	10,434,375 (57.1)
Dual-eligible, No. (%)	3,931,929 (16.8)	2,842,509 (17.1)	3,026,003 (16.6)
Age, mean (sd)	78.7 (7.7)	78.8 (8.0)	78.8 (8.1)
Hospital characteristics			
No. of hospitals	3,703	3,527	3,441
Safety-net hospital, No. (%)	817 (22.1)	787 (22.3)	771 (22.4)
Hospital bed size, mean (sd)	214 (211)	220 (219)	225 (224)
Profit status[a], No. (%)			
For profit	908 (24.5)	837 (23.7)	824 (23.9)
Nonprofit	1,751 (47.3)	1,683 (47.7)	1,736 (50.5)
Public	427 (11.5)	355 (10.1)	328 (9.5)
Other	1,100 (29.7)	930 (26.4)	884 (25.7)
Teaching hospital, No. (%)	831 (22.4)	801 (22.7)	825 (24.0)
Rural hospital, No. (%)	1,028 (27.8)	981 (27.8)	947 (27.5)
Geographic region, No. (%)			
Northeast	580 (15.7)	544 (15.4)	522 (15.2)
Midwest	823 (22.2)	794 (22.5)	785 (22.8)
South	1,588 (42.9)	1,526 (43.3)	1,487 (43.2)
West	712 (19.2)	663 (18.8)	647 (18.8)

Note: The Pre-ACA period began on January 1, 2007 and ended on March 31, 2010. The implementation period began on April 1, 2010 and ended on September 30, 2012. The HRRP penalty period began on October 1, 2012 and ended on September 30, 2015.

ACA = Affordable Care Act; HRRP = Hospital Readmissions Reduction Program

© 2018 Chaiyachati KH et al. *JAMA Network Open*.

eTable 2. Risk-Adjusted Trends, Differences in Trends Between Black And White Patients by Time Periods and Difference-in-Differences in Trends Between Time Periods, by Hospitals' Safety-Net Status Among the HRRP Targeted Conditions[a]

Clinical conditions	Safety-net hospitals [n=11,237,242 discharges]				Non-safety-net hospitals [n=46,999,814 discharges]			
	White Patients	Black Patients	Difference (95% CI)	P value	White Patients	Black Patients	Difference (95% CI)	P value
**HRRP-targeted conditions [n=7,864,250]**								
Time periods								
Pre-ACA	-0.04	+0.02	+0.06 (-0.01 to +0.13)	0.08	-0.02	+0.01	+0.03 (-0.01 to +0.07)	0.18
HRRP implementation	-0.20	-0.34	-0.14 (-0.22 to -0.06)	<0.01	-0.21	-0.30	-0.08 (-0.13 to -0.03)	<0.01
HRRP penalty	-0.01	+0.01	+0.01 (-0.07 to +0.10)	0.74	-0.02	+0.01	+0.03 (-0.02 to +0.08)	0.25
Time period comparisons ("difference-in-differences")								
Pre-ACA vs. HRRP implementation			-0.21 (-0.34 to -0.07)	<0.01			-0.11 (-0.19 to -0.03)	<0.01
HRRP implementation vs. HRRP penalty			+0.16 (+0.01 to +0.30)	0.03			+0.11 (+0.02 to +0.20)	0.01
HRRP penalty vs. pre-ACA			-0.05 (-0.15 to +0.05)	0.34			0.00 (-0.06 to +0.06)	0.94

Note: HRRP-targeted conditions included acute myocardial infarction, heart failure, and pneumonia. The Pre-ACA period began on January 1, 2007 and ended on March 31, 2010. The implementation period began on April 1, 2010 and ended on September 30, 2012. The HRRP penalty period began on October 1, 2012 and ended on September 30, 2015.

[a] All estimates were adjusted for patient characteristics (i.e., age, sex, 30 comorbidities defined by Medicare's hospital readmission risk adjustment, and Medicare/Medicaid dual-eligible status), hospital characteristics (i.e., number of beds, profit status, teaching hospital status as indicated by having an allopathic or osteopathic residency program, rural location of hospital, and Census-designated region), and incorporated hospital fixed effects.

ACA = Affordable Care Act; HRRP = Hospital Readmissions Reduction Program.

© 2018 Chaiyachati KH et al. *JAMA Network Open*.

eTable 3. Risk-Adjusted Trends, Differences in Trends Between Black and White Patients by Time Periods and Difference-in-Differences in Trends Between Time Periods, by Hospitals' Safety-Net Status Among Conditions Not Targeted by the HRRP[a]

Clinical conditions	Safety-net hospitals [n=11,237,242 discharges]				Non-safety-net hospitals [n=46,999,814 discharges]			
	White Patients	Black Patients	Difference (95% CI)	P value	White Patients	Black Patients	Difference (95% CI)	P value
**Non-targeted conditions [n=50,372,806]**								
Time periods								
Pre-ACA	-0.02	0.00	+0.01 (-0.01 to +0.04)	0.40	-0.02	-0.02	+0.01 (-0.01 to +0.02)	0.36
HRRP implementation	-0.13	-0.23	-0.10 (-0.13 to -0.06)	<0.001	-0.13	-0.17	-0.04 (-0.06 to -0.02)	<0.001
HRRP penalty	-0.01	+0.04	+0.05 (+0.01 to +0.08)	<0.01	0.00	0.00	0.00 (-0.01 to +0.02)	0.81
Time period comparisons ("difference-in-differences")								
Pre-ACA vs. HRRP implementation			-0.11 (-0.16 to -0.06)	<0.001			-0.05 (-0.08 to -0.02)	<0.01
HRRP implementation vs. HRRP penalty			+0.14 (+0.09 to +0.20)	<0.001			+0.04 (+0.01 to +0.08)	0.02
HRRP penalty vs. pre-ACA			+0.03 (0.00 to +0.07)	0.08			+0.01 (-0.03 to +0.02)	0.67

Note: Non-targeted conditions included any condition other than acute myocardial infarction, heart failure, and pneumonia. The Pre-ACA period began on January 1, 2007 and ended on March 31, 2010. The implementation period began on April 1, 2010 and ended on September 30, 2012. The HRRP penalty period began on October 1, 2012 and ended on September 30, 2015.

[a] All estimates were adjusted for patient characteristics (i.e., age, sex, 30 comorbidities defined by Medicare's hospital readmission risk adjustment, and Medicare/Medicaid dual-eligible status), hospital characteristics (i.e., number of beds, profit status, teaching hospital status as indicated by having an allopathic or osteopathic residency program, rural location of hospital, and Census-designated region), and incorporated hospital fixed effects.

ACA = Affordable Care Act; HRRP = Hospital Readmissions Reduction Program.

© 2018 Chaiyachati KH et al. *JAMA Network Open*.
